# Are waiting times for hospital admissions affected by patients' choices and mobility?

**DOI:** 10.1186/1472-6963-11-170

**Published:** 2011-07-15

**Authors:** Ånen Ringard, Terje P Hagen

**Affiliations:** 1 Department of Health Management and Health Economics University of Oslo Norway

**Keywords:** Patient choice, elective treatment, waiting times, Norway

## Abstract

**Background:**

Waiting times for elective care have been considered a serious problem in many health care systems. A topic of particular concern has been how administrative boundaries act as barriers to efficient patient flows. In Norway, a policy combining patient's choice of hospital and removal of restriction on referrals was introduced in 2001, thereby creating a nationwide competitive referral system for elective hospital treatment. The article aims to analyse if patient choice and an increased opportunity for geographical mobility has reduced waiting times for individual elective patients.

**Methods:**

A survey conducted among Norwegian somatic patients in 2004 gave information about whether the choice of hospital was made by the individual patient or by others. Survey data was then merged with administrative data on which hospital that actually performed the treatment. The administrative data also gave individual waiting time for hospital admission. Demographics, socio-economic position, and medical need were controlled for to determine the effect of choice and mobility upon waiting time. Several statistical models, including one with instrument variables for choice and mobility, were run.

**Results:**

Patients who had neither chosen hospital individually nor bypassed the local hospital for other reasons faced the longest waiting times. Next were patients who individually had chosen the local hospital, followed by patients who had not made an individual choice, but had bypassed the local hospital for other reasons. Patients who had made a choice to bypass the local hospitals waited on average 11 weeks less than the first group.

**Conclusion:**

The analysis indicates that a policy combining increased opportunity for hospital choice with the removal of rules restricting referrals can reduce waiting times for individual elective patients. Results were robust over different model specifications.

## 1. Background

Waiting times for elective surgery have been, and still are, an important health policy concern in many advanced industrial countries. Policymakers have over the last twenty years increasingly addressed the question of how to tackle the problem [1]. Both supply and demand policies have been introduced in an attempt to meet the challenge posed by excessive waiting times [2]. Initiatives on the demand side include: prioritizing patients according to need and providing treatment only to medium and high risk patients; encouraging private health insurance cover to reduce the demand for public treatment and increase the demand for private treatment; and allowing patients greater opportunities for choosing their hospital [1]. The latter type of reform has been introduced in Sweden, Norway, Denmark, The Netherlands, England, Germany, France, and Belgium [3-6]. In Norway, the reform, which came into effect in 2001, also meant a removal of rules restricting referrals to providers within a single district, thereby creating a competitive referral system for elective care [7,8].

There has, in periods, been a lot of political interest in the potential benefits of patient choice. Claims about choice being a vehicle for improving accessibility have also been made on theoretical grounds [9]. Despite this optimism, recent reviews of the patient choice literature in the UK, Europe and USA, conclude that there is still little evidence of greater choice having such effects [4,5,10]. A hospital-level analysis of the effect of choice on waiting times in the UK concluded that more choice was significantly associated with shorter waiting times. The quantitative effect, however, was modest. The same study also provided evidence indicating that more choice can have the adverse effect of boosting waiting times [11]. At the patient level, an evaluation of the London Patient Choice Project showed a reduction in waiting times for all patients, including those outside the project [6].

Patient mobility (i.e. geographical movement by patients across administrative boundaries to other hospitals) was first described in a study of waiting times for elective surgery in Canada [12]. The study also included a taxonomy of causes of mobility. The causes identified were: patients' choice of hospital, general practitioner (GP) induced mobility, and structurally caused mobility.

Here, the conceptual separation of patient choice and patient mobility is used as a framework for evaluating patient choice reforms. We first distinguished between a dimension that describe the decision (if the choice is made by the individual patient or by others, for example the GP) and a dimension that describe the result of this decision (if the result imply geographical mobility or not). Secondly, we combined the two dimensions to form a typology with four different alternatives: one which implied neither choice nor mobility (i.e. not bypassing the local hospital); one with individual choice but without mobility; one without individual choice but with mobility; and one with both individual choice and mobility. The non-choice and mobility alternative, would be due to either a GP's decision or influenced by structural features of the health care system (e.g. hospital specialization).

Prior to the reform Norwegian policymakers blamed long waiting times for elective care both to the lack of patient choice and administrative borders creating barriers for patients moving to hospital facilities in other counties. Consequently it was believed that shorter waiting times could be obtained through a policy combining an individual right for patients to choose hospital with the removal of county border barriers [13]. The article aims to shed light on whether the introduction of patient choice and the increased opportunity for geographical mobility has contributed to reduce waiting times for elective patients at patient level. Whether changes in waiting time at individual level affect the overall waiting time of the system is another question and a question that will not be discuss. It will, however, to some extent depend on the utilization of the general capacity of the system. If this increases due to increased patient mobility, the overall waiting times should be reduced [6].

## 2. Institutional setting

In Norway, long waiting times became an important part of the perception of a general "health care crisis" during the 1980s and 1990s. Prolonged waiting times were not a new phenomenon, but it was not until the 1985 parliamentary election campaign that it was considered politically unacceptable [14]. Independent reports at the time estimated that more than 100 000 patients were waiting for in-patient care, and that the number had been increasing since the beginning of the 1970s [15].

**Figure 1:**
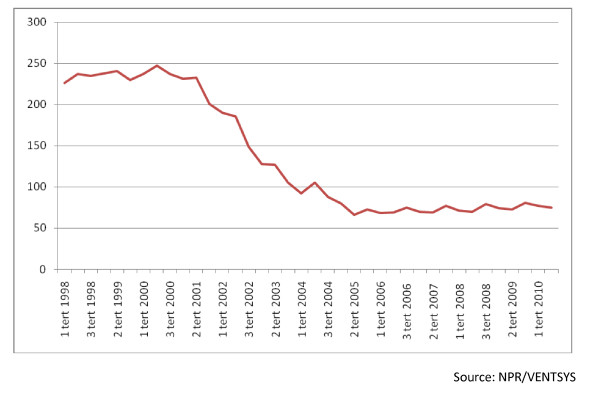
**Average waiting times in days for somatic treatment 1998-2010**.

Figure 1 depicts the development in waiting times for elective in-patient and outpatient treatment between 1998 and 2006 across the five Norwegian health care regions. The figure confirms the description of long average waiting times for elective patients in the 90s. In the period from 1998 to 2000, the average waiting time was close to 250 days. From 2000 the average waiting time dropped markedly. From 2005 it has been stable at about 70 days.

In the 1980s and early 1990s the favoured answer was to increase the capacity of hospitals without increasing total health care expenditure. Thus, efficiency was to be improved through organizational and other non-financial measures [14]. In 1997, activity-based financing (ABF) of hospitals was introduced. Recent research has shown that the introduction of ABF and the subsequent expansion of hospital budgets were both important factors in reducing waiting times for elective treatment [16]. Another, often cited cause of reduced waiting times was the "cleaning up" of hospital waiting lists from 2000 to 2002 [17].

In the latter part of the 1990s, a new initiative was taken, this time related to the demand side. The Patients' Rights Act, which came into effect on January 1st 2001, implied that patients were given an explicit right to decide at which hospital to receive elective treatment and the removal of geographical limitations on choice. All previous geographical barriers to mobility were removed so patients got the opportunity to choose a hospital anywhere in the country. The right to choose hospital was extended in 2004 to also include private hospitals under contract with a Regional Health Authority.

## 3. Methods

### Research design

The major difficulty within empirical studies on hospital choice has been how to identify patient choice from administrative data. Moreover, administrative data normally lack pre-hospitalization information about patients. In an attempt to overcome this problem, a cross-sectional survey covering questions related to personal hospital choices was carried out among Norwegian elective patients in 2004 (questionnaire included as additional file 1).

To allow for generalization, the study sample was drawn using a two-stage procedure. In the first stage, 20 hospitals were randomly selected among all Norwegian hospitals in order to assure representativeness regarding both hospital size and geographical location. In the second stage, 4000 elective patients were drawn from the registers of the 20 hospitals. The Patient Choice Pilot Project evaluation (1994-1996) found both patient choice and mobility to be rare phenomena [18]. Consequently, it was decided to increase the proportion of mobile patients (i.e. patients not treated locally) in the study. The total sample contained 3000 mobile patients and 1000 that had been treated at the local hospital. Due to the study design, the data used in the following analyses has been weighted to reflect the universe consisting of all Norwegian elective patients.

The participants received a self-administered questionnaire in the mail during the summer and autumn of 2004 [19]. The questions asked were all related to a particular treatment episode, which took place between the autumn of 2003 and spring of 2004. 664 questionnaires were returned unopened, and were removed from the sample. After one reminder, completed questionnaires were obtained from 1678 patients, giving a response rate of close to 50%. The questionnaire information was then combined with administrative data about the same treatment episode from the Norwegian Patient Register (NPR).

Participants were asked whether they had chosen the hospital where they had been treated or not. NPR provided information about the participant's home municipality and place of treatment (hospital). This made it possible to determine if the patient had been treated locally (non-mobile) or had bypassed the local hospital (mobile). The survey data and the register data were then combined to operationalise the typology of choice and mobility presented in the introduction. Thus, individual patients were categorised into four groups: Group 1-Patients that had neither chosen nor bypassed the local hospital; Group 2-Patients that had chosen not to bypass the local hospital; Group 3-Patients that had not chosen but still had moved; Group 4-patients that had both chosen and moved.

### The dependent variable

To measure the actual waiting time for each patient, information provided by NPR was used. The waiting time variable was constructed using the difference between the date of hospital admission for the particular treatment episode and the date the hospital received the letter of referral. The latter date was assumed to be a good proxy for the referral date. Private hospitals were, at the time of the survey, not obliged to report the referral date to the NPR. 78 privately treated participants were therefore excluded. We further suspect that some extreme waiting times were due to measurement errors and therefore set maximum waiting time to four and a half years (1650 days). This excluded 4.5 percent of the sample.

### Explanatory variables

The analysis of choice and mobility was based upon the assumptions that all patients had the wish to be treated without delay, but that there were variations in their opportunities or capacity to pursue this aim. Moreover, it was assumed that it was the individual patient's expectation of shorter waiting times that was the prime motivator for the decision to choose hospital. Differences in motivation can be caused by a particular illness (e.g. dementia) or other idiosyncratic factors. Furthermore it was assumed that those who were mobile had better opportunities to travel than those who did not, due to for instance illness, duties at home, lack of time etc. According to this it seems fair to assume that group 4 patients will have shorter waiting times than group 1 patients who had neither chosen nor moved. The remaining groups, which are less clear cut, will probably face waiting times somewhere between group 1 and 4. Patients in the second group had made a deliberate decision to be treated locally, perhaps because cost of waiting (e.g. travel expenses, being away from home) for the local hospital had been perceived to be less than costs associated with travelling elsewhere. Patients in the third group had not chosen the hospital but have still received treatment elsewhere. Thus, someone, like the referring doctor, must have been acting on their behalf. If this was the case, it is the referring physician who has possessed information both about the patient' preferences and differences in waiting times, and has been willing to use this in a beneficial way for the patient.

Other variables were included as controls [20-23]. We included five socioeconomic variables: *gender, age, personal income, educational level and participant's area of residence *(for details, see the technical appendix). The patient's need for health care services was measured by *self-assessed health, GP-utilization *and *hospital utilization*. Two variables related to the particular treatment episode were also included. *The condition treated *was based on the ICD-10 chapter classification. Chapter 21 (factors influencing health status and contact with health services) is the reference category. In addition, a dummy for *type of treatment *was created to distinguish between patients who had been treated for a medical condition (non-surgical) and those treated for a surgical condition. Finally, a dummy variable of the *year of treatment *was included in the model. The last three variable vectors were used to construct fixed-effect regression models. The fixed effect models means that we utilize variations within these groups to estimate the effects of patient choice and mobility.

### Statistics and estimation method

Since it is the wish to be treated without delay, an omitted variable, that drives choice and mobility, it can be argued that variables describing choice and mobility are endogenous and need to be instrumented [24]. We therefore supplemented the OLS models with fixed effects with a two stage least square model (2SLS) where instruments for choice and mobility were included. Reliable instruments for the four variables that described choice and mobility were however difficult to find and we were forced to instrument a dummy variable that took the value of 1 if the patient either had chosen or was mobile, otherwise it took the value of 0 (no choice, no mobility). Age, gender, personal income, educational level, self-assessed health, type of treatment (surgical vs. non-surgical) and distance to local hospitals were used as instruments. The validity of the instruments was tested by the method recommended by Dranove and Wehner [25]. We correlated the residual from the first stage regression and our instruments and found no significant correlation for personal income, educational level and type of treatment. As a consequence, these variables were excluded from the second stage regression. Overidentification was tested by a Basmann test and rejected (F = 2.61, p = 0.05) [26]. The dependent variable was skewed and therefore log-transformed in two of the specifications. Statistical analyses were performed by SAS, version 9.2. Final regressions were made by "proc reg" and "proc syslin".

The project, including the survey, was approved by the local regional medical ethics committee (REK Sør) in December 2005 (reference S- 05344).

## 4. Results

Waiting times for elective hospital treatment were, as seen in Figure 1, still substantial. Table 1 shows the average waiting times of the participants in the study according to their condition described by main diagnoses of the ICD-10 system.

**Table 1:** Average waiting times for elective treatment according to main diagnoses groups (ICD10), 2003-2004

Chapter	ICD10 chapter	N	Mean wts	S.D
				

2	Neoplasm and diseases of the blood and blood-forming organs	116	374.6	531.9

4	Endocrine, nutritional and metabolic diseases	40	489.0	599.9

6	Diseases of the nervous system	122	251.4	355.0

7	Diseases of the eye and adnexa	79	264.4	389.5

8	Diseases of the ear and mastoid process	56	453.8	490.5

9	Diseases of the circulatory system	92	261.0	345.8

10	Diseases of the respiratory system	64	260.6	361.8

11	Diseases of the digestive system	79	252.5	437.8

12	Diseases of the skin and subcutaneous tissue	64	327.3	457.6

13	Diseases of the musculoskeletal system and connective tissue	248	308.4	445.5

14	Diseases of the genitourinary system	71	216.1	338.7

15	Pregnancy, childbirth and the puerperium	38	130.4	144.9

17	Congenital malformations, deformations and chromosomal abnormalities	48	587.4	573.3

18	Symptoms, signs and abnormal clinical and laboratory findings, not elsewhere classified	78	234.1	285.0

19	Injury, poisoning and certain other consequences of external causes	117	157.4	203.8

21	Factors influencing health status and contact with health services	270	218.7	333.6

				

	**Total**	**1596**	**278.8**	**405.9**

Patients waited on average 278.8 days from the time of referral to hospital admission. The higher waiting time in table 1 compared to Figure 1, reflects both differences in data source and registration practice. Figure 1 is based on hospital reports on waiting time from referral to starting point of a treatment period, and is taken from the National system for waiting time-VENTSYS. Table 1, on the other hand, reflects waiting time from referral to a specific treatment (that can be within a treatment period), and is based on information from NPR. There were large differences in waiting times for different conditions. Patients treated for conditions associated with pregnancy, childbirth and the puerperium (Chapter 15) waited about 130 days, while those treated for congenial malformations, deformations, and chromosome abnormalities (Chapter 17) waited an average of 587 days.

Table 2 contains weighted descriptive information on survey participants. On average, the participants were approximately 46 years old at the time of the study and had about four years of formal tertiary education. Sixty-two per cent reported good or very good general health prior to the treatment episode.

**Table 2:** Descriptive statistics for the participants included in the analyses*

Variable	N	Min	Max	Mean	S.D
					

Wts-Waiting time	1674	1	1650	299	431

Log(wts)	1674	-9.21	7.40	3.86	3.90

					

Age	1674	0	96	46.1	22.95

Gender (female = 1)	1674	0	1	0.45	0.50

Distance to local hospital (km)	1671	2	353	31.8	46.3

Education (years after primary school)	1490	0	17	3.9	3.1

Income	1552	1	6	3.35	1.47

					

Health status	1604	0	1	0.62	0.49

Number of visits to GP	1601	0	1	0.37	0.48

Number of hospital stays	1518	0	1	0.35	0.48

					

Non-choice & non-mobile patients	1626	0	1	0.46	0.49

Choice & non-mobile patients	1626	0	1	0.20	0.40

Non-choice & mobile patients	1626	0	1	0.17	0.38

Choice & mobile patients	1626	0	1	0.13	0.34

					

Year of treatment (2004 = 1)	1674	0	1	0.63	0.48

					

Type of treatment (surgical = 1)	1674	0	1	0.23	0.42

* Weighted results.

Table 3 shows the results from the regression analyses. In Model 1 (non-logged dependent variable) socio-demographic variables and patients' need for hospital services were included on the right hand side in addition to fixed effects for year, medical conditions (ICD-10) and type of treatment. Women had on average 24 days longer waiting time than men. Patients who reported good or very good health waited approximately three months less than those with poor health. Finally, patients who had visited their GP more than five times during the past 12 months reduced their waiting time of almost three months compared to those with fewer visits.

**Table 3:** Multivariate analysis of waiting times for elective care (estimates with standard error in parentheses)

Variable:	Model 1	**Sig**.	Model 2	**Sig**.	Model 3	**Sig**.	Model 4	**Sig**.
	**Estimate (S.E)**		**Estimate (S.E)**		**Estimate (S.E)**		**Estimate (S.E)**	

Age	-0.31 (0.63)		-0.35 (0.64)		-0.01 (0.01)		-0.00 (0.00)	

Gender	23.66 (12.54)	*	24.27 (12.53)	*	0.15 (0.15)		0.30 (0.13)	**

Distance to local hospital	-0.18 (0.26)		-0.14 (0.26)		0.00 (0.00)		0.00 (0.00)	

Education	-5.50 (4.24)		-3.74 (4.29)		-0.11 (0.07)	*	-	

Income	-9.31 (9.54)		-10.98 (9.55)		-0.08 (0.12)		-	

								

Health status	-99.07 (28.76)	****	-90.19 (28.96)	***	-0.51 (0.32)		0.24 (0.40)	

Number of visits to GP	-89.67 (27.31)	****	-85.95 (27.30)	***	-0.03 (0.29)		0.20 (0.31)	

Number of hospital stays	0.83 (26.15)		5.59 (26.23)		0.16 (0.30)		0.52 (0.31)	*

								

Non-choice & non-mobile	-		0.00		0.00		0.00	

Choice & non-mobile	-		-50.60 (30.12)	*	-0.24 (0.45)		-	

Non-choice & mobile	-		-65.80 (33.58)	**	-1.29 (0.32)	****	-	

Choice & mobile	-		-78.19 (36.04)	**	-0.62 (0.33)	*	-	

IV for choice/mobility	-		-		-		-5.36 (1.46)	****

								

Year 2003	76.58 (24.77)	***	77.04 (24.88)	***	-0.45 (0.28)		-0.34 (0.27)	

Year 2004	0.00		0.00		0.00		0.00	

ICD10 chapter 2	307.39(51.84)	****	304.16 (51.88)	****	1.71 (0.59)	***	4.33 (1.52)	***

ICD10 chapter 4	351.46 (77.57)	****	347.25 (77.49)	****	2.26 (0.51)	****	4.92 (1.64)	****

ICD10 chapter 6	69.70 (53.65)		80.17 (53.83)		-0.70 (0.77)		1.66 (1.51)	

ICD10 chapter 7	106.32 (59.83)	*	112.60 (59.82)	*	1.02 (0.57)	*	3.63 (1.55)	**

ICD10 chapter 8	240.73 (71.45)	****	239.69 (71.43)	****	1.10 (1.10)		3.83 (1.61)	**

ICD10 chapter 9	53.65 (52.34)		46.92 (52.38)		1.17 (0.66)	*	3.80 (1.51)	**

ICD10 chapter 10	104.20(63.39)		101.55 (63.30)		1.38 (0.52)	***	4.12 (1.55)	***

ICD10 chapter 11	75.02 (56.06)		67.08 (56.12)		1.70 (0.49)	****	4.32 (1.53)	****

ICD10 chapter 12	82.97 (63.19)		72.68 (63.35)		1.30 (0.58)	**	3.97 (1.57)	**

ICD10 chapter 13	124.00 (39.46)	***	126.27 (39.49)	***	1.02 (0.47)	**	3.61 (1.47)	**

ICD10 chapter 14	76.00 (59.14)		72.22 (59.15)		1.18 (0.51)	**	3.73 (1.54)	**

ICD10 chapter 15	-21.30(68.32)		-26.36 (68.40)		-0.75 (1.26)		1.87 (1.60)	

ICD10 chapter 17	274.38 (84.51)	****	276.53 (84.42)	****	2.28 (0.57)	****	5.10 (1.66)	****

ICD10 chapter 18	-30.48 (61.28)		-33.45 (61.24)		0.49 (0.81)		3.11 (1.55)	**

ICD10 chapter 19	-53.81 (54.18)		-49.94 (54.16)		-1.42 (0.87)		1.18 (1.51)	

ICD10 chapter 21	0.00		0.00		0.00		2.61 (1.46)	*

Medical treatment	83.71 (28.57)	***	73.27 (28.80)	***	0.60 (0.28)	**	-	

Surgical treatment	0.00		0.00		0.00		-	

								

Estimation procedure	OLS with fixed effects		OLS with fixed effects		OLS with fixed effects		2SLS with fixed effects	

Number of observations	1274		1274		1348		1361	

-2LogLikelihood	19202		19169		8048			

R2							0,07	

****sig. = 0.001, *** sig. = 0.01, **sig. = 0.05, *sig. = 0.10								

In model 2 (non-logged dependent variable) the choice and mobility variables were included. There were small changes in the effects of the socio-demographic and variables describing health status. The estimates of the fixed-effect variables also remained stable. The dummy variables describing choice and mobility (where non-choice and non-mobile patients are the reference category) showed the expected pattern. Patients belonging to the reference category faced the longest waiting time. On average, they waited seven weeks longer than patients who themselves had chosen the local hospital. Those who had not chosen but had still moved waited almost nine and a half weeks less than the reference group. Finally, patients who individually had chosen a hospital and moved experienced a shorter wait of about 11 weeks relative to the reference category.

Model 3 contains the same explanatory variables as model 2 but with a logged dependent variable to handle the problem of skewness. The associations between the demographic and enabling factors and waiting time were, with the exception of education, insignificant. The variables describing health status lost their significance. The effects of the choice and mobility dummies were still important to explain differences in waiting time as patients belonging to the reference category (non-choice and non-mobile patients) had longer waiting time than those who has either chosen or moved. The results did however indicate that mobility was more important in explaining differences in waiting time than choice, as the variable describing choice and non-mobility became non-significant.

Model 4 (logged dependent variable) contained the same explanatory variables as the previous two models, but the explanatory variables describing choice and mobility have now been instrumented. The estimates of the demographic, enabling and need variables are similar to those of model 3. The instrument variable describing choice or mobility was highly significant and indicates that choice and mobility were important predictors of waiting time.

## 5. Discussion

Prolonged waiting times for elective treatment have long been considered a serious problem of the Norwegian health care system. Overall waiting times have, due to both supply and demand reforms, dropped significantly over recent years. Despite the positive development, many patients still face substantial waiting times. The analysis provides support for claiming that a policy of combining patient choice of hospital with the removal of geographical restrictions on referrals may contribute to the reduction in waiting times for individual elective patients. The main results of the empirical analyses were found to be robust over different statistical model specifications. Patients who chose to move hospitals benefit the most. They spend about 11 weeks less, on average, waiting for hospital treatment than those who neither chose nor moved. The results corroborate with the results of a few previous empirical analyses, like The London Patient Choice Project. There, a reduction in waiting times for all patients was found after choice was introduced, including also for those who did not participate in the project [6].

Compared with previous analyses this study has some methodological advantages. By combining register data with information from a specially designed survey of patients, it has been possible to measure empirically both the individual patient's hospital choice and the subsequent mobility/non-mobility. This has not been possible in studies based upon register data [18]. Compared with general population studies, the current study offers another advantage. To study patients, and ask them about their experiences relating to a particular treatment episode, brings us closer to actually investigating revealed preferences (what people do) than stated preferences (what people say they do) [27]. Finally, our sample was drawn in a manner that permits generalization, and tests have been conducted to ensure that the study sample does not diverge from the patient population on important characteristics such as gender and factors related to the hospital stay. The only exception is that the study participants on average are about two year younger than the Norwegian patient population as a whole.

The main limitation in our study is the number of non-responders. This is, however, not a problem confined to this particular study; most surveys of hospital patients in Norway struggle to reach high response rates [28]. Research suggests that the main reason for non-response is that many patients still are, when invited to participate in a survey, too sick or weak to fill in a questionnaire [29]. A selection bias can therefore not entirely be ruled out. The study design does not allow us to draw firm causal inferences although the instrument variable approach comes close to that. In order to do this an alternative study design (e.g. a pre-post design with a control group) would have been necessary. Finally, one methodological limitation has to be noted for the identification strategy of the IV-approach (table 3, model 4). As reliable instruments were hard to find, the results of the model should be interpreted with some care.

The results of these analyses ought to make patients and policy makers more receptive to the potential benefits of hospital choices. For patients, the immediate implication of the study is that if one chooses to bypass the local hospital, waiting time for hospital admission may be significantly reduced. Another interesting finding is that even if a patient allows somebody else to decide on his/her behalf, the waiting time will also be reduced, given that the patient is willing to travel to an alternative hospital. This may for instance occur because the referring physician has used their knowledge about spare hospital capacity to the benefit of the patient. From the patient perspective, the least beneficial option, when it comes to waiting, is not to choose and to end up at the local hospital. From the perspective of policymakers, the main implication is that the introduction of choice and the removal of geographical boundaries (county borders) both contribute to a reduction in waiting time for patients. Another interesting result is the negative association between health status and waiting time. One interpretation could be that patients with better health finds the prospect of travelling (i.e. to become mobile) less daunting, and therefore in turn are able to benefit from the existence of shorter waiting times elsewhere. Moreover, the analyses indicate that reduced waiting times can be achieved without jeopardizing the aim of equal geographical and social access to hospital care. Further insight might also be gained by looking into the role of referring physicians in choice and mobility decisions.

## 6. Conclusion

There is still relatively limited empirical evidence on the impact of patient choice (4-6; 10). One possible direction for future research could be to refine the choice/non-choice dichotomy used here. This line of research could benefit from integrating concepts such as "shared decision-making" [30], and "patient involvement/participation" [31]. A second possible direction would be to expand the analyses of choice and mobility to explore the impact upon other health related outcomes like quality [32].

## Competing interests

### Financial competing interests

In the past five years have you received reimbursements, fees, funding, or salary from an organization that may in any way gain or lose financially from the publication of this manuscript, either now or in the future? No (ÅR and TPH).

Do you hold any stocks or shares in an organization that may in any way gain or lose financially from the publication of this manuscript, either now or in the future? No (ÅR and TPH).

Do you hold or are you currently applying for any patents relating to the content of the manuscript? Have you received reimbursements, fees, funding, or salary from an organization that holds or has applied for patents relating to the content of the manuscript? No (ÅR and TPH).

Do you have any other financial competing interests? No (ÅR and TPH).

### Non-financial competing interests

Are there any non-financial competing interests (political, personal, religious, ideological, academic, intellectual, commercial or any other) to declare in relation to this manuscript?

No (ÅR and TPH).

## Authors' contributions

ÅR designed and carried out the patient choice study, performed the analysis of data and drafted the manuscript. TPH participated in the design of the study and contributed substantially to the statistical analysis. Both authors read and approved the final manuscript.

## Technical appendix

**Variable name:** Waiting times

**Data source:** Norwegian patient register (NPR)

**Description:** Number of days waited by the patient from the reception of the referral letter at the hospital to the actual treatment

**Coding:** Continuous variable

**Variable name:** Age

**Data source:** Norwegian patient register (NPR)

**Coding:** Continuous variable

**Variable name:** Gender

**Data source:** Norwegian patient register (NPR)

**Coding:** 0 = Male, 1 = Female

**Variable name:** Distance to local hospital

**Data source:** Register data

**Description:** Number of kilometres from centre of home municipality to the local hospital

**Coding:** Continuous variable

**Variable name:** Education

**Data source:** Norwegian choice and mobility survey (the CM-survey)

**Description:** Number of years education after primary school

**Coding:** Continuous variable

**Variable name:** Income

**Data source:** Norwegian choice and mobility survey (the CM-survey)

**Description:** Income in NOK

**Coding:** Continuous variable 1 = less than 60,000 (NOK) 6 = 500,000 or more

**Variable name:** Health status

**Data source:** Norwegian choice and mobility survey (the CM-survey)

**Description:** Dummy variable

**Coding:** 0 = Bad/Not so good 1 = Good/Very good

**Variable name:** Number of visits to GP in previous 12 months

**Data source:** Norwegian choice and mobility survey (the CM-survey)

**Description:** Dummy variable

**Coding:** 0 = Five or fewer visits 1 = More than five

**Variable name:** Number of hospital stays in previous 12 months

**Data source:** Norwegian choice and mobility survey (the CM-survey)

**Description:** Dummy variable

**Coding:** 0 = Only one stay 1 = More than one stay

**Variable name:** Patient choice and mobility

**Data source:** Combined information from the CM-survey and NPR

**Description:** Dummy variable

**Coding:** 0 = Non-choice & non-mobility

1 = Choice & non-mobility

2 = Non-choice & mobility

3 = Choice & mobility

**Variable name:** Year of investigation

**Data source:** Norwegian choice and mobility survey

**Description:** Dummy variable

**Coding:** 0 = 2003 1 = 2004

**Variable name:** Type of medical treatment received

**Data source:** Norwegian patient register (NPR)

**Description:** Dummy variable

**Coding:** 0 = Medical treatment 1 = Surgical treatment

**Variable name:** Main condition treated in the hospital episode under consideration

**Data source:** Norwegian patient register (NPR)

**Description:** Dummy variable based upon ICD-10 chapters (see Table 1 for the names of the individual chapters):

**Coding:** 0 = Other condition (Ch 21)

1 = Chapter 2

2 = Chapter 4

3 = Chapter 6

4 = Chapter 7

4 = Chapter 8

4 = Chapter 9

4 = Chapter 10

4 = Chapter 11

4 = Chapter 12

4 = Chapter 13

4 = Chapter 14

4 = Chapter 15

4 = Chapter 17

4 = Chapter 18

4 = Chapter 19

## Pre-publication history

The pre-publication history for this paper can be accessed here:

http://www.biomedcentral.com/1472-6963/11/170/prepub

## Supplementary Material

Additional file 1 **The questionnaire used in the patient choice and mobility survey**. Questionnaire (in Norwegian). Click here for file

## References

[B1] Siciliani L Hurst J Explaining waiting time variations for elective surgery across OECD countries 2003 Paris: OECD

[B2] Mossialos E Le Grand J Health care and cost containment in the European Union 1999 Aldershot: Ashgate

[B3] Magee H Davies LJ Coulter A Public views on healthcare performance indicators and patient choice 2003 96 Journal of the Royal Society of Medicine 338 342 10.1258/jrsm.96.7.338 PMC539537 12835446

[B4] Thomson S Dixon A Choices in health care: the European experience 2006 11 3 Journal of Health Services Research and Policy 167 171 10.1258/135581906777641703 16824264

[B5] Winblad U Spongberg Ringard Å Magnussen J, Vrangbæ K, Saltman RB Meeting rising public expectations: the changing role of patients and citizens Nordic health care systems--Recent Reforms and Current Policy Challenges 2009 McGraw-Hill/Open University Press

[B6] Dawson D Gravelle H Jacobs R Martin S Smith PC The effects of expanding patient choice of provider on waiting times: evidence from a policy experiment Health Economics 2007 16 113 128 10.1002/hec.1146 16888753

[B7] Kjønstad A Molven O The development of patients' rights in Norway The Norwegian Health Care System 1999 Oslo: Centre for Medical Studies, Moscow

[B8] Kjønstad A Helserett--pasienter og helsearbeiders rettstilling 2007 Oslo: Gyldendal Akdemisk [In Norwegian-Health care law-patients and practitioners legal status]

[B9] Le Grand J The Other Invisible Hand: Delivering public services through choice and competition 2007 Princeton: Princeton University Press

[B10] Fotaki M Roland M Boyd A McDonald R Scheaff R Smith L What benefit will choice bring to patients? Literature review and assessment of implications 2008 13 3 Journal of Health Services Research and Policy 178 184 10.1258/jhsrp.2008.007163 18573768

[B11] Siciliani L Martin S An empirical analysis of the impact of choice on waiting times Health Economics 2007 16 763 779 10.1002/hec.1205 17238226

[B12] Tessier G Contrandiopoulos A Dionne G Patient mobility for elective surgical interventions Social Science and Medicine 1985 20 1307 1312 10.1016/0277-9536(85)90385-5 4023765

[B13] Ot.prp. nr 12 (1998-99): Lov om pasientrettigheter (pasientrettighetsloven) Helse og omsorgsdepartementet 13.11.1998 [In Norwegian-White Paper no. 12 (1998-1999): The Patients' Right Act]

[B14] Skaset M Schiøtz A Reformtid og markedsgløtt: Det offentlige helsevesen etter 1985 Folkets helse-landets styrke 1850-2003 2003 Oslo: Universitetsforlaget [In Norwegian-Times of reform and marketization: The Public Health Service since 1985]

[B15] Berg O Fra politikk til økonomikk 2005 Oslo: Den norske legeforening [In Norwegian-From politics to econometrics]

[B16] Hagen TP Kaarboe OM The Norwegian hospital reform of 2002: central government takes over ownership of public hospitals Health Policy 2006 76 3 320 333 10.1016/j.healthpol.2005.06.014 16099530

[B17] Sundar T Ryddesjau etter reformen fjerner ventelister Tidsskrift for den Norske Lægeforening 2002 22 122 [In Norwegian-Clearing of waiting lists after the reform]

[B18] Jørgensen S Vanskelige valg? Evaluering av fritt sykehusvalg i helseregion II 1994-1996 1997 Trondheim: SINTEF Unimed [In Norwegian-Tough decisions? Evaluation of hospital choice in health region II 1994-1996]

[B19] Christensen Ø Hem KG Fritt sykehusvalg i Norge 2004 Oslo, SINTEF Helse [In Norwegian-Hospital choice in Norway]

[B20] Andersen R A behavioural model of families' use of health services 1968 Chicago: Center for Health Administration Studies, University of Chicago

[B21] Kurz RS Wolinsky FD Who picks the hospital: Practitioner or patient? 1985 March/April Hospital and Health Service Administration 95 106 10270716

[B22] Porell FW Adams EK Hospital choice models: A review and assessment of their utility for policy impact analysis Medical Care Research and Review 1995 52 158 195 10.1177/107755879505200202 10154559

[B23] Andersen R Revisiting the behavioral model and access to medical care: does it matter? Journal of Health and Social Behaviour 1995 36 1 10 10.2307/2137284 7738325

[B24] Wooldridge JM Introductory Econometrics-A modern approach 2006 Mason, Ohio, USA: Thomson

[B25] Dranove D Wehner P Physician-induced demand for childbirths Journal of Health Economics 1994 13 61 73 10.1016/0167-6296(94)90004-3 10134439

[B26] Basmann RL On Finite Sample Distributions of Generalized Classical Linear Identifiability Test Statistics Journal of the American Statistical Association 1960 55 650 659 10.2307/2281588

[B27] Brouwer W van Exel J Hermans B Stoop A Should I stay or should I go? Waiting lists and cross-border care in The Netherlands Health Policy 2003 63 289 298 10.1016/S0168-8510(02)00120-3 12595128

[B28] Garrat A Helgeland J Metodedokumentasjon for nasjonal undersøkelse i 2006 2007 Oslo: Nasjonalt kunnskapssenter for helsetjenesten [In Norwegian-Documentation report for the 2006 patient satisfaction study]

[B29] Guldvog B Hofoss D Pettersen KI Ebbesen J Rønning OM. PS-RESKVA-pasienttilfredshet i sykehus Tidsskrift for den Norske Lægeforening 1998 118 386 391 [In Norwegian-Patient satisfaction in hospitals] 9499727

[B30] Coulter A Whatever happened to patients' decision-making? Health Expectations 2002 5 185 186 10.1046/j.1369-6513.2002.00190.x 12199657 PMC5060156

[B31] Thompson AGH The meaning of patient involvement and participation in health care consumption: A taxonomy Social Science & Medicine 2007 64 1297 1310 10.1016/j.socscimed.2006.11.002 17174016

[B32] Donabedian A An introduction to quality assurance in health care 2003 Oxford: Oxford University Press

