# Supplementary material for: Are waiting times for hospital admissions affected by patients' choices and mobility?
Source: BMC Health Serv Res. 2011 Jul 15;11:170. doi: 10.1186/1472-6963-11-170 (PMC3160356; doi:10.1186/1472-6963-11-170)
Supplement: Additional file 1 — The questionnaire used in the patient choice and mobility survey. Questionnaire (in Norwegian). [file 1472-6963-11-170-S1.PDF]

< inst> <pas>

SINTEF Helse

Postadresse:
Pb 124, Blindern, 0314 Oslo/
7465 Trondheim

Telefon:
22 06 73 00 (Oslo)
73 59 30 00 (Trondheim)
Telefaks:
22 06 79 09 (Oslo)
73 59 63 61 (Trondheim)

Foretaksregisteret:
NO 948 007 029 MVA

Oslo, juni 2004

Spørreundersøkelse om fritt sykehusvalg

SINTEF Helse gjennomfører etter oppdrag fra Helsedepartementet en spørreundersøkelse om fritt sykehusvalg. Ordningen med at pasienter ved sykehusene fritt kan velge hvilket sykehus de kan behandles ved har nå eksistert i over to år. Men vi kjenner faktisk ikke til hvem, eller hvor mange, som benytter denne retten. Vi må derfor spørre pasientene selv.

Du har i løpet av 2004/2003 vært pasient ved < sykehus >. Ved hjelp av sykehuset har vi plukket ut noen pasienter som får tilsendt et spørreskjema. Vi vil be deg om å fylle ut dette spørreskjemaet, og returnere det til SINTEF Helse i den vedlagte svarkonvolutten så snart som mulig. Porto er allerede betalt. Det er frivillig om du vil delta i undersøkelsen, men for å få så gode data som mulig er det viktig at flest mulig svarer.

Ved hjelp av sykehuset vil vi knytte sammen opplysningene i skjemaet med opplysninger fra sykehusets register om din behandling, sykdom, alder, kjønn og kommune. Ved å returnere skjemaet gir du samtidig tillatelse til at vi kobler sammen disse opplysningene ved hjelp av løpenummeret på skjemaet. Når foresatte besvarer spørreskjemaet på vegne av mindreårige pasienter, gir de på samme måte tillatelse til at sykehusene foretar koblingene for disse pasientene.

Skjemaene returneres anonymt til SINTEF Helse, og vi vil ikke få opplyst navn eller andre opplysninger som kan identifisere den enkelte pasient. Prosjektet avsluttes i 21. august 2004. Frem til denne datoen vil man kunne trekke seg fra prosjektet. Etter denne datoen vil alle personopplysninger bli slettet eller anonymisert. Ingen opplysninger som fremkommer i sluttrapporten vil kunne tilbakeføres til enkeltindivider. Alle involverte i prosjektet har taushetsplikt. SINTEF Helse vil behandle alle data konfidensielt. Prosjektet er meldt til Personvernombudet for forskning, Norsk samfunnsvitenskapelig datatjeneste AS.

Skjemaene blir lest maskinelt, det er derfor viktig å sette kryss inni boksene.

Har du noen spørsmål rundt undersøkelsen kan du kontakte:
Karl-Gerhard Hem, tlf. 22 06 74 55, e-post: hem@sintef.no eller Øyvind Christensen tlf. 22 06 75 60.

Vi takker på forhånd for hjelpen!

Med vennlig hilsen
for SINTEF Helse

Arne H. Eide
Forskningssjef

Karl-Gerhard Hem
Forsker

Før sykehusbehandlingen

1. Valgte du selv det sykehuset du ble behandlet på?

Ja ☐ Nei ☐ *(Hvis nei, gå til spørsmål 9).*

2. Hvis ja, hvordan ble valget foretatt?

Jeg valgte sykehus helt på egen hånd. ☐

Det var legen min som i praksis valgte sykehuset for meg. ☐

Valget ble foretatt av min lege og meg selv i fellesskap. ☐

Valget ble foretatt på tross av råd fra min lege. ☐

Annen grunn ☐

3. Kjente du til ordningen med fritt sykehusvalg før du var hos legen?

Ja ☐ Nei ☐ *(Hvis nei, gå til spørsmål 8).*

4. Hvordan ble du klar over ordningen med fritt sykehusvalg? *(Sett om nødvendig flere kryss).*

Annonse i dagspressen ☐
Informasjon fra Internett ☐
Opplysningstlf. for fritt sykehusvalg ☐

Kampanjeinformasjon fra sykehus ☐
Privat informasjonsformidling ☐
Informasjon fra venner/bekjente ☐

Brev fra sykehuset ☐
Informasjon fra familie/pårørende ☐
Informasjon fra min lege ☐

Annet: ________________ ☐

5. Nedenfor har vi listet opp noen kilder til informasjon om "Fritt sykehusvalg". Hvor viktige var disse i forbindelse med ditt valg av sykehus? *(Sett ett kryss for hver linje).*

	Svært viktig	Viktig	Lite viktig	Ikke viktig
Annonse i dagspressen	☐	☐	☐	☐
Informasjon fra Internett	☐	☐	☐	☐
Opplysningstelefonen for fritt sykehusvalg	☐	☐	☐	☐
Kampanjeinformasjon fra sykehuset	☐	☐	☐	☐
Privat informasjonsformidling	☐	☐	☐	☐
Informasjon fra venner/bekjente	☐	☐	☐	☐
Informasjon fra familie/pårørende	☐	☐	☐	☐
Informasjon fra min lege	☐	☐	☐	☐
Annen informasjonskilde:___________	☐	☐	☐	☐

6. Nedenfor har vi listet opp ulike forhold som mange mener er viktige når man skal velge sykehus. Hvor viktige var disse for deg da du valgte sykehus? *(Sett ett kryss for hver linje).*

	Svært viktig	Viktig	Lite viktig	Ikke viktig
Sykehuset ligger nærmest der jeg bor	☐	☐	☐	☐
Ventetiden var kort ved dette sykehuset	☐	☐	☐	☐
Sykehuset har et godt rykte	☐	☐	☐	☐
Sykehuset har godt og moderne utstyr	☐	☐	☐	☐
Sykehuset har høy kompetanse på området	☐	☐	☐	☐
Opplysninger om sykehuskvalitet på Internett	☐	☐	☐	☐
Min lege anbefalte sykehuset	☐	☐	☐	☐
Jeg har god erfaring fra tidligere kontakt	☐	☐	☐	☐
Jeg har dårlig erfaring fra tidligere kontakt	☐	☐	☐	☐

T

Hva synes du om?

6. Hvor tilfreds var du med den informasjonen du på forhånd mottok om ordningen med fritt sykehusvalg?

Svært tilfreds	Tilfreds	Utilfreds	Svært utilfreds
☐	☐	☐	☐

7. Hvor tilfreds var du med den informasjonen legen din gav deg om ordningen med fritt sykehusvalg?

Svært tilfreds	Tilfreds	Utilfreds	Svært utilfreds
☐	☐	☐	☐

8. Kunne du tenke deg å velge det samme sykehuset igjen?

Ja ☐ Nei ☐

9. Egenandelen ved reiser i forbindelse med fritt sykehusvalg er i dag kr 220 hver vei, til sammen kr. 440. La oss tenke oss at egenandelen nå øker.

a. Ville du fortsatt benyttet deg av fritt sykehusvalg dersom egenandelen økte fra kr 220 hver vei til kr 400 hver vei, til sammen kr. 800?

Ja ☐ Nei ☐

b. Ville du fortsatt benyttet deg av fritt sykehusvalg dersom egenandelen økte fra kr 220 hver vei til kr 600 hver vei, til sammen kr. 1200?

Ja ☐ Nei ☐

c. Ville du fortsatt benyttet deg av fritt sykehusvalg dersom egenandelen økte fra kr 220 hver vei til kr 800 hver vei, til sammen kr. 1600?

Ja ☐ Nei ☐

10. Hva mener du om ventetiden fra du ble henvist til sykehuset til du ble lagt inn?

Altfor lang	For lang	Passe lang	For kort	Altfor kort
☐	☐	☐	☐	☐

T

T

11. Hvor tilfreds var du med den informasjonen du fikk da du ble lagt inn på sykehuset?

Svært tilfreds	Tilfreds	Utilfreds	Svært utilfreds
☐	☐	☐	☐

Reisen til sykehuset

12. Hvordan kom du deg til sykehuset?
(Sett om nødvendig flere kryss).

☐ Privatbil ☐ Buss
☐ Tog ☐ Fly
☐ Taxi ☐ Båt/Ferge

13. Måtte du overnatte på grunn av reisen?

Ja ☐ Nei ☐

14. Hvor lang tid brukte du hjemmefra til sykehuset?

Under 1 time ☐
Ca. 1-2 timer ☐
Ca. 3-4 timer ☐
Ca. 5-8 timer ☐
Mer enn 8 timer ☐

15. Betalte for du reisen selv?

Ja ☐ Jeg reiste på rekvisisjon ☐

16. Hva betalte du i egenandel?

[_ _ _] kroner.

17. Hva omtrent kostet reisen fram og tilbake totalt (før eventuelle refusjoner)?

[_ _ _ _ _] kroner.

18. Hadde du frikort i 2003?

Ja ☐ Nei ☐

T

19. Hvor viktig er det for deg at du har muligheten til selv å kunne velge sykehus?

Svært viktig	Viktig	Lite viktig	Uvesentlig
☐	☐	☐	☐

20. Vil du, på bakgrunn av dine erfaringer, benytte deg av din rett til å velge sykehus igjen?

Ja ☐ Nei ☐

Bakgrunnsopplysninger

Opplysningene nedenfor skal brukes til å undersøke om ulike pasientgrupper har forskjellige erfaringer med sine sykehusbehandling, og det er derfor viktig at du besvarer spørsmålene.

21. Hvor ofte har du vært behandlet eller undersøkt hos lege eller primærlege de siste 12 månedene?

Bare denne ene gangen	2-5 ganger	Mer enn 5 ganger
☐	☐	☐

22. Hvor mange ganger i løpet av de siste 12 månedene har du vært innlagt på sykehus?

Bare denne ene gangen	2-5 ganger	Mer enn 5 ganger
☐	☐	☐

23. Stort sett, vil du si at helsen din er:

Dårlig	Ikke helt god	God	Svært god
☐	☐	☐	☐

T

24. Hva gjør du til daglig?
(Sett om nødvendig flere kryss).

Er yrkesaktiv ☐
Er hjemmearbeidende ☐
Er uføretrygdet ☐
Er sykemeldt/på attføring ☐
Mottar sosialhjelp ☐
Er pensjonist ☐
Er under utdanning/student ☐
Annet:______________ ☐

25. Hvor stor er din inntekt? *(Brutto årsinntekt, inkludert trygd/pensjon, før skatt og fradrag er fratrukket)*

0-60.000 ☐
60-100.000 ☐
100-200.000 ☐
200-300.000 ☐
300-400.000 ☐
400-500.000 ☐
Over 500.000 ☐

26. Hvor mange år har du gått på skole/tatt utdanning *etter* at du var ferdig med grunnskole?

Antall år: [_ _]

(Obligatorisk grunnskole = 0, alle årene etter grunnskolen legges sammen)

27. Hvor mange personer bor i din husstand?

Jeg bor alene ☐
Vi er 2 personer ☐
Vi er 3 personer eller flere ☐

Vennligst legg skjemaet i den vedlagte konvolutten, og postlegg konvolutten. Porto er allerede betalt.

Tusen takk for hjelpen!
